# Supplementary material for: An atlas of the human liver diurnal transcriptome and its perturbation by hepatitis C virus infection
Source: Nat Commun. 2024 Aug 29;15:7486. doi: 10.1038/s41467-024-51698-8 (PMC11362569; doi:10.1038/s41467-024-51698-8)
Supplement: Supplementary file 1 — Supplementary Information [file 41467_2024_51698_MOESM1_ESM.pdf]

Supplementary Information

An atlas of the human liver diurnal transcriptome and its perturbation by hepatitis C virus infection

Atish Mukherji[1,*], Frank Jühling[1,*], Yogy Simanjuntak[1], Emilie Crouchet[1], Fabio Del Zompo[1], Yuji Teraoka[2], Alexandre Haller[3], Philippe Baltzinger[3], Soumith Paritala[4], Fahmida Rasha[4], Naoto Fujiwara[4], Cloé Gadenne[1], Nevena Slovic[1], Marine A. Oudot[1], Sarah C. Durand[1], Clara Ponsolles[1], Catherine Schuster[1], Xiaodong Zhuang[5,6], Jacinta Holmes[7], Ming-Lun Yeh[8], Hiromi Abe-Chayama[9], Mathias Heikenwälder[10,11], Angelo Sangiovanni[12], Massimo Iavarone[12], Massimo Colombo[13], Steven K. H. Foung[14], Jane A. McKeating[5,15], Irwin Davidson[3], Ming-Lung Yu[8,16], Raymond T. Chung[17], Yujin Hoshida[4], Kazuaki Chayama[18,19,20], Joachim Lupberger[1,§,#], and Thomas F. Baumert[1,21,22, 23,§,#]

[1]University of Strasbourg, Institute of Translational Medicine and Liver Diseases (ITM), Inserm UMR_S1110, Strasbourg, France.
[2]Department of Gastroenterology, National Hospital Organization Kure Medical Center, Hiroshima, Japan.
[3]Department of Functional Genomics and Cancer, Institut de Génétique et de Biologie Moléculaire et Cellulaire (IGBMC), CNRS/INSERM/University of Strasbourg, Illkirch, France.
[4]Department of Internal Medicine, University of Texas Southwestern Medical Center, Dallas, USA.
[5]Nuffield Department of Medicine, University of Oxford, Oxford OX3 7FZ, UK.
[6]Institute of Immunity & Transplantation, Division of Infection & Immunity, UCL, Pears Building, Rowland Hill St, London NW3 2PP, UK.
[7]University of Melbourne, St Vincent's Hospital, Melbourne, Australia.
[8]Hepatobiliary Division, Department of Internal Medicine, School of Medicine and Hepatitis Research Center, College of Medicine, and Center for Liquid Biopsy and Cohort Research, Kaohsiung Medical University Hospital, Kaohsiung Medical University, Kaohsiung 80708, Taiwan.
[9]Center for Medical Specialist Graduate Education and Research, Hiroshima, Japan.
[10]Division of Chronic Inflammation and Cancer, German Cancer Research Center (DKFZ), Heidelberg, Germany.
[11]M3 Research Center, Tübingen, Germany and Cluster of Excellence iFIT (EXC 2180) "Image-Guided and Functionally Instructed Tumor Therapies," Eberhard-Karls University of Tübingen, Tübingen, Germany.
[12]Division of Gastroenterology and Hepatology, Fondazione IRCCS Cà Granda Ospedale Maggiore Policlinico, Milan, Italy.
[13]EASL International Liver Foundation, Geneva, Switzerland.
[14]Department of Pathology, Stanford University School of Medicine, California 94305, USA.
[15]Chinese Academy of Medical Sciences Oxford Institute, University of Oxford, UK.
[16]School of Medicine and Doctoral Program of Clinical and Experimental Medicine, College of Medicine and Center of Excellence for Metabolic Associated Fatty Liver Disease, National Sun Yat-sen University, Kaohsiung, Taiwan.
[17]Gastrointestinal Division, Hepatology and Liver Center, Massachusetts General Hospital, Boston, MA 02114, USA.
[18]Collaborative Research Laboratory of Medical Innovation, Graduate School of Biomedical and Health Sciences, Hiroshima University, Hiroshima, Japan.
[19]RIKEN Center for Integrative Medical Sciences, Yokohama, Japan.
[20]Hiroshima Institute of Life Sciences, Hiroshima, Japan.
[21]Gastroenterology and Hepatology Service, Strasbourg University Hospitals, France.
[22]Institut Universitaire de France, Paris, France.
[23]IHU Strasbourg, France

[#]Correspondence to: Prof. Thomas F. Baumert, MD, and Dr. Joachim Lupberger, Ph.D., Inserm U1110, University of Strasbourg, 3 Rue Koeberlé, F-67000 Strasbourg, France. Phone: +33368853703, email: thomas.baumert@unistra.fr , joachim.lupberger@unistra.fr
[*]A.M. and F.J. contributed equally to this work. §J.L. and T.F.B. having jointly supervised this work.

<u>Supplementary Figure 1:</u> Representative images of hematoxylin-eosin (HE) and human hepatocyte-specific CK8-18 staining of non-infected control human liver chimeric mouse (HCLM) livers. Wide field HE images revealed conserved lobular architecture of engrafted human hepatocytes and absence of stress response. Residual murine hepatocytes (histologically chracterized by a smaller size and more eosinophillic staining that their human engrafted cells) are present in the perivascular regions. Scale bars: 500 µm.

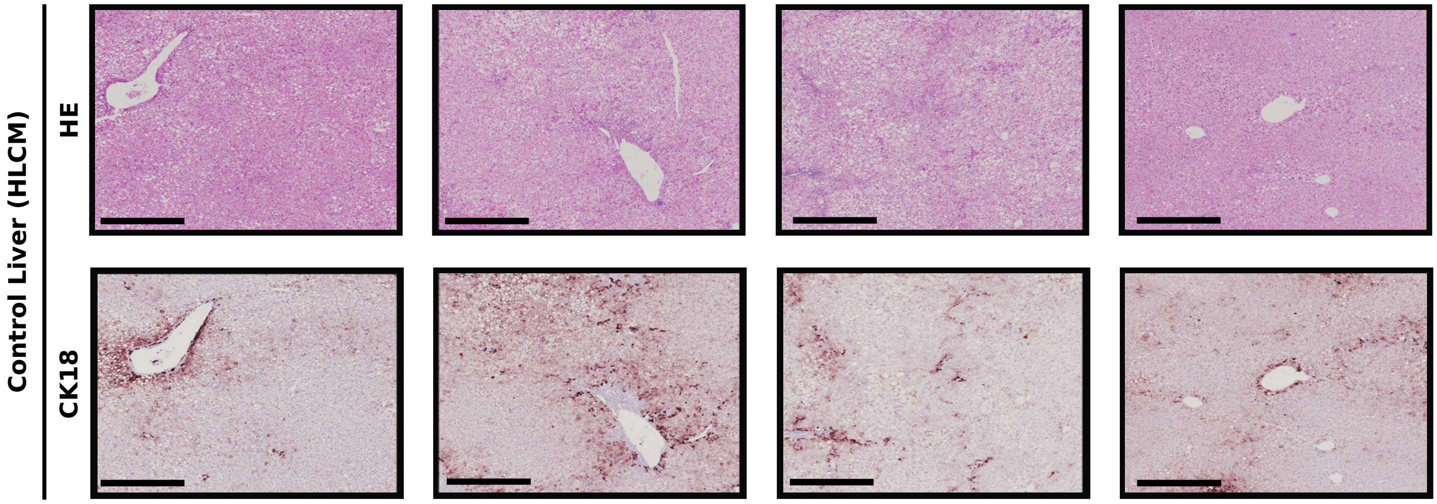

<u>Supplementary Figure 2:</u> Unsupervised clustering of human hepatocytes in non-infected control humanized liver chimeric mouse (HCLM) livers and CC genes from two independent experiments: **a** Series 1 and **b** Series 2. 3 HLCM were used in each timepoint. Samples highlighted with bold font in **a** were selected for further analysis. Sample S76790_ZT16 (Series 1 in **a**; indicated in red) was excluded due to low coverage and low humanization level (3 HLCM/timepoint, n=18 HLCM/experiment). Source data are provided as a Source Data file.

a

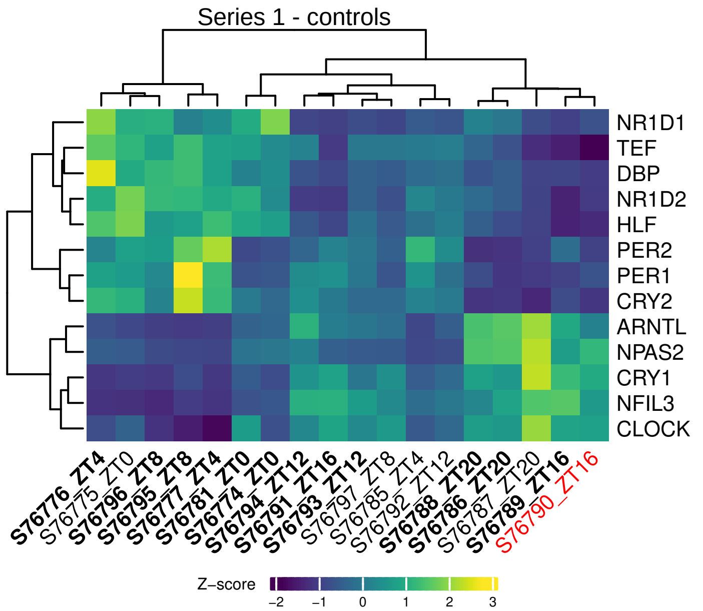

b

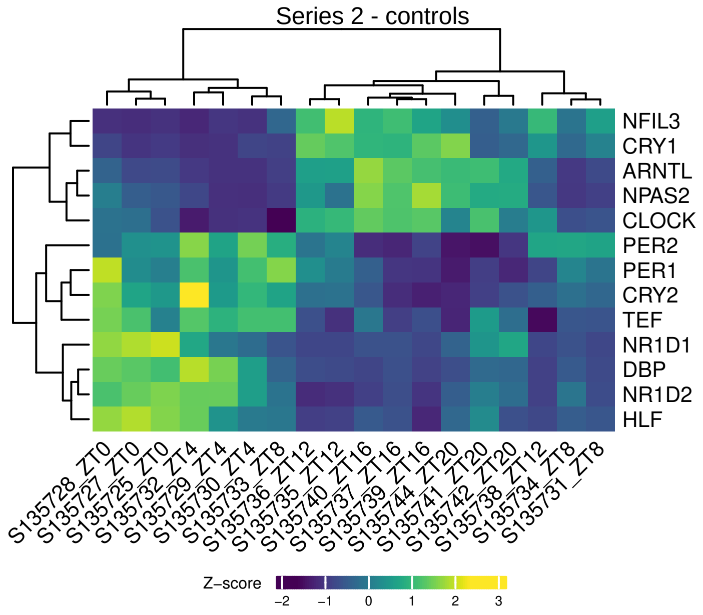

<u>Supplementary Figure 3:</u> a Mean expression pattern of core CC genes and CC output regulators in human (red) and mouse (green) cells in non-infected control HLCM livers (n=5 HLCM/ timepoint). The curves are shown as normalized DESeq2 read counts. Bars represent SD. Source data are provided as a Source Data file. **b** Mean expression of CC genes in human and mouse cells in non-infected control HLCM livers (n=5 HLCM/ timepoint) and WT mice livers as published in Koike et al. Science 2012 (n=2/column). Source data are provided as a Source Data file.

a

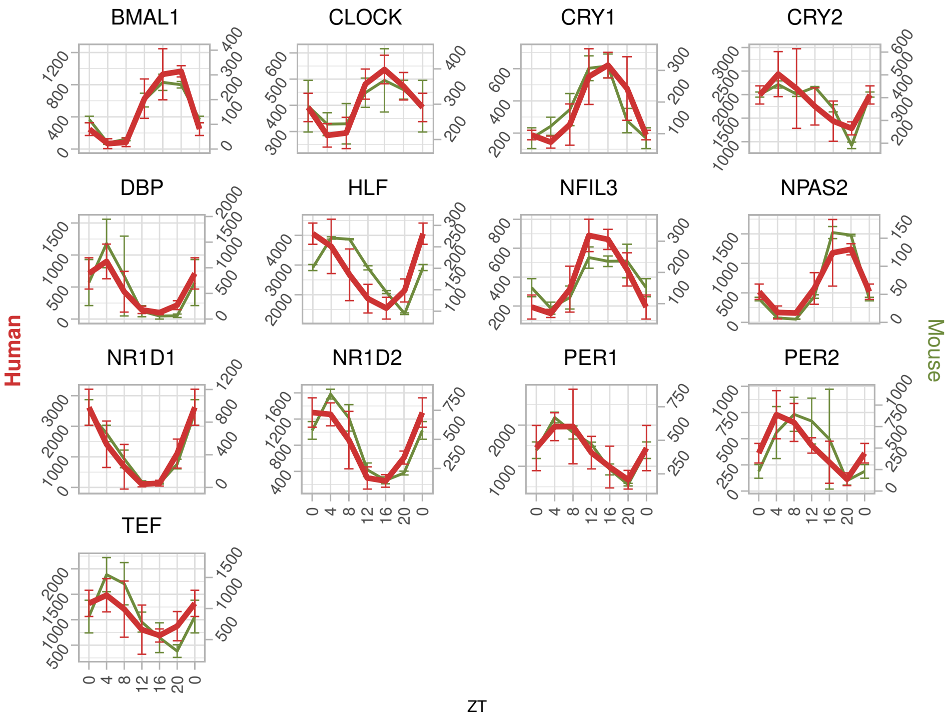

b

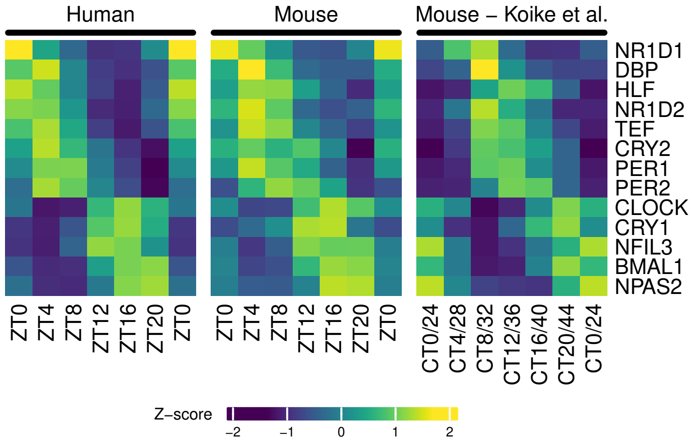

<u>Supplementary Figure 4</u>: a dryR genes with shared and/or unique temporal gene expression pattern in human or mouse cells in non-infected control HLCM livers (Fig. 1c), and genes with rhythmic expression pattern in wild type mice as published in Koike et al. Science 2012. **b** Genes with shared and/or unique temporal gene expression pattern in human or mouse cells in non-infected control HLCM livers (Fig. 1c), and genes with rhythmic expression in post-mortem human liver (Talamanca et al. Science 2023). Hypergeometric tests reveal non-significant overlaps.

a

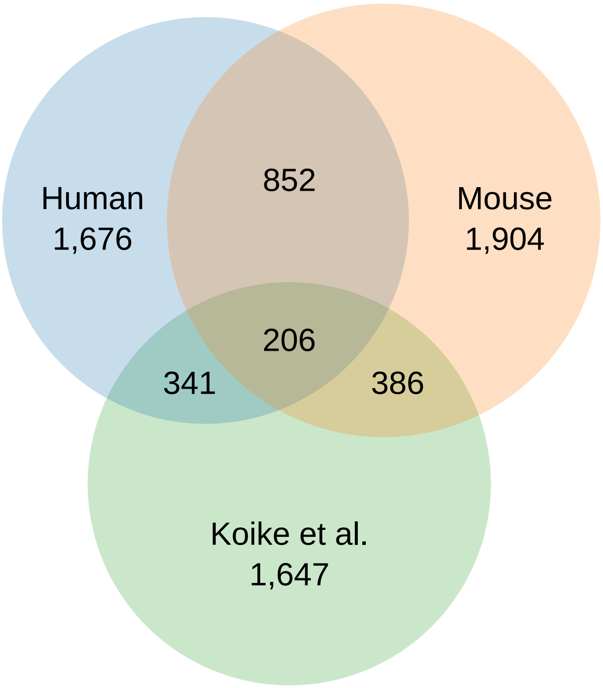

b

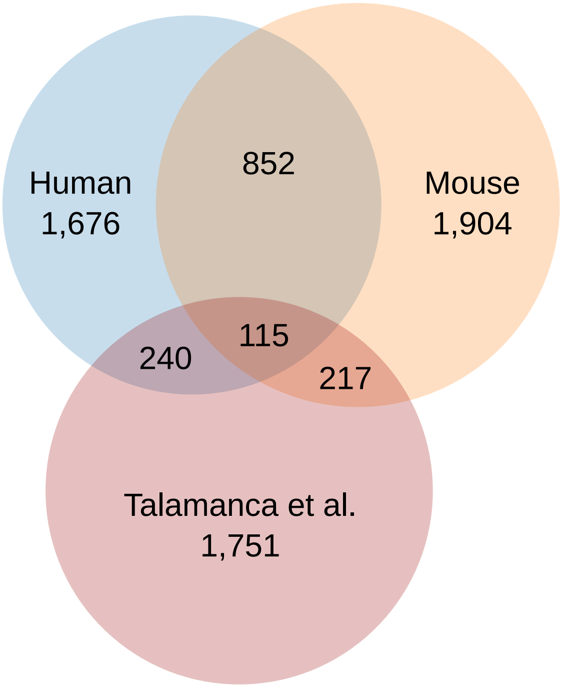

Supplementary Figure 5: KEGG and BIOCARTA pathways significantly (FDR<0.05) enriched for genes displaying temporal variations in human hepatocytes in HLCM livers as listed in (Fig. 1C) and their expression in non-infected control mice. Similar pathways **a** show overlapping (±1 ZT step) of peak enrichment scores comparing human and WT mice maximum enrichment scores. **b** Pathways significantly (FDR<0.05) enriched for genes displaying distinct (separated by more than 1 ZT) variations in human hepatocytes in HLCM as listed in (Fig. 1C) and their expression in non-infected control mice. BC: BIOCARTA, and KE: KEGG. N=5 HLCM/timepoint. ZT0: represented twice in each panel to maintain conformity. Source data are provided as a Source Data file.

a

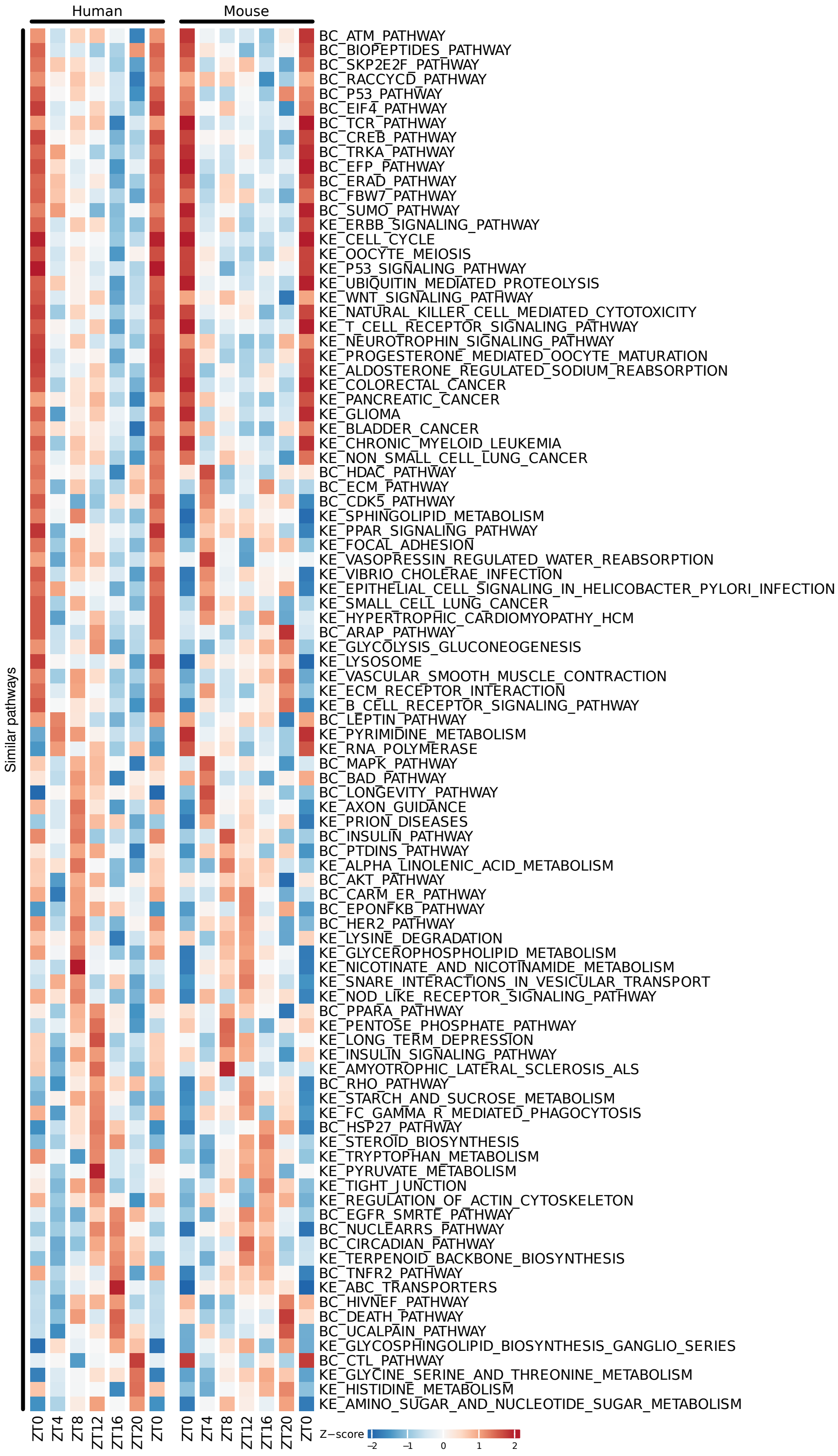

b

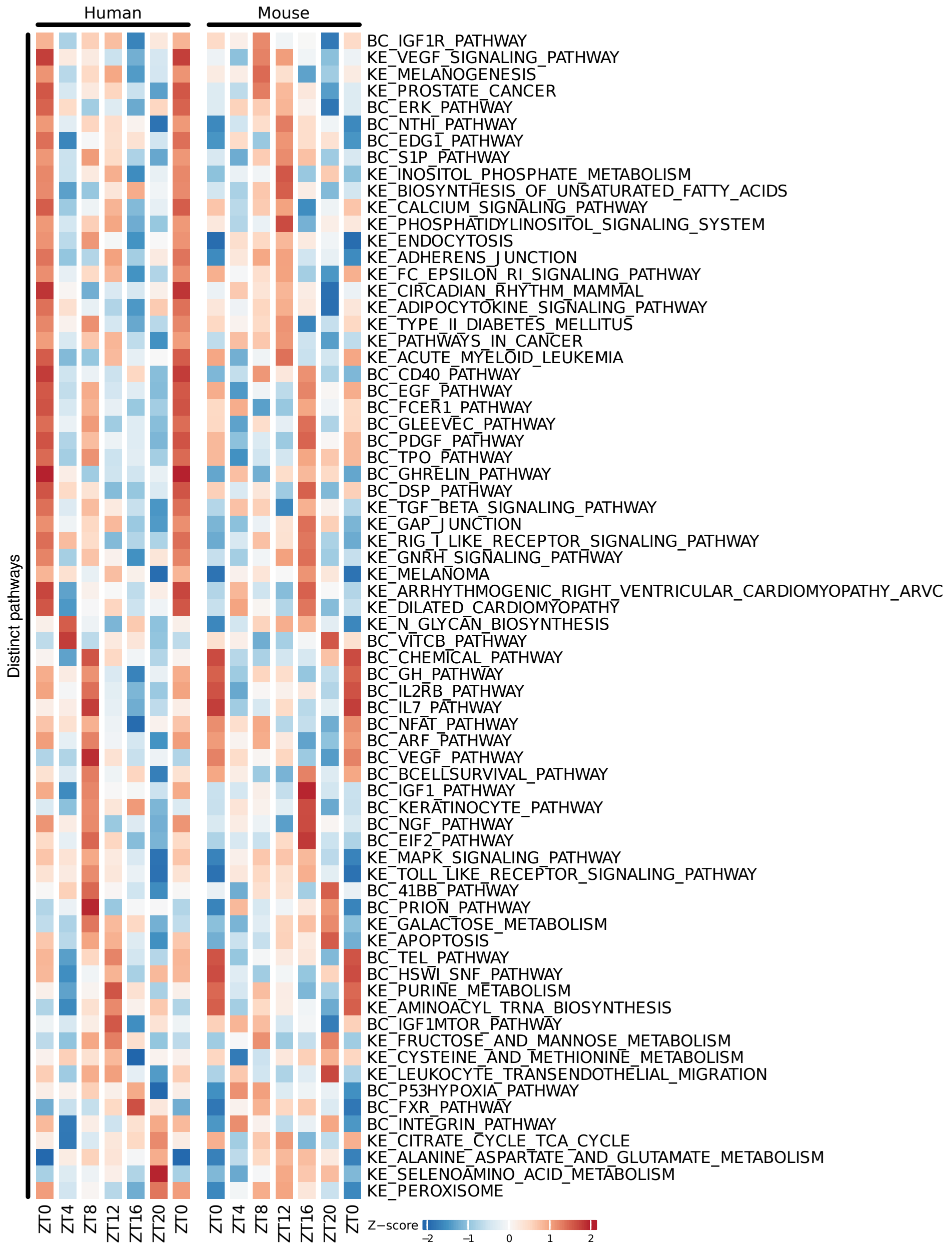
Human
Mouse
Distinct pathways
BC_IGF1R_PATHWAY
KE_VEGF_SIGNALING_PATHWAY
KE_MELANOGENESIS
KE_PROSTATE_CANCER
BC_ERK_PATHWAY
BC_NTHI_PATHWAY
BC_EDG1_PATHWAY
BC_S1P_PATHWAY
KE_INOSITOL_PHOSPHATE_METABOLISM
KE_BIOSYNTHESIS_OF_UNSATURATED_FATTY_ACIDS
KE_CALCIUM_SIGNALING_PATHWAY
KE_PHOSPHATIDYLINOSITOL_SIGNALING_SYSTEM
KE_ENDOCYTOSIS
KE_ADHERENS_JUNCTION
KE_FC_EPSILON_RI_SIGNALING_PATHWAY
KE_CIRCADIAN_RHYTHM_MAMMAL
KE_ADIPOCYTOKINE_SIGNALING_PATHWAY
KE_TYPE_II_DIABETES_MELLITUS
KE_PATHWAYS_IN_CANCER
KE_ACUTE_MYELOID_LEUKEMIA
BC_CD40_PATHWAY
BC_EGF_PATHWAY
BC_FCER1_PATHWAY
BC_GLEEVEC_PATHWAY
BC_PDGF_PATHWAY
BC_TPO_PATHWAY
BC_GHRELIN_PATHWAY
BC_DSP_PATHWAY
KE_TGF_BETA_SIGNALING_PATHWAY
KE_GAP_JUNCTION
KE_RIG_I_LIKE_RECEPTOR_SIGNALING_PATHWAY
KE_GNRH_SIGNALING_PATHWAY
KE_MELANOMA
KE_ARRHYTHMOGENIC_RIGHT_VENTRICULAR_CARDIOMYOPATHY_ARVC
KE_DILATED_CARDIOMYOPATHY
KE_N_GLYCAN_BIOSYNTHESIS
BC_VITCB_PATHWAY
BC_CHEMICAL_PATHWAY
BC_GH_PATHWAY
BC_IL2RB_PATHWAY
BC_IL7_PATHWAY
BC_NFAT_PATHWAY
BC_ARF_PATHWAY
BC_VEGF_PATHWAY
BC_BCELLSURVIVAL_PATHWAY
BC_IGF1_PATHWAY
BC_KERATINOCYTE_PATHWAY
BC_NGF_PATHWAY
BC_EIF2_PATHWAY
KE_MAPK_SIGNALING_PATHWAY
KE_TOLL_LIKE_RECEPTOR_SIGNALING_PATHWAY
BC_41BB_PATHWAY
BC_PRION_PATHWAY
KE_GALACTOSE_METABOLISM
KE_APOPTOSIS
BC_TEL_PATHWAY
BC_HSWI_SNF_PATHWAY
KE_PURINE_METABOLISM
KE_AMINOACYL_TRNA_BIOSYNTHESIS
BC_IGF1MTOR_PATHWAY
KE_FRUCTOSE_AND_MANNOSE_METABOLISM
KE_CYSTEINE_AND_METHIONINE_METABOLISM
KE_LEUKOCYTE_TRANSENDOTHELIAL_MIGRATION
BC_P53HYPOXIA_PATHWAY
BC_FXR_PATHWAY
BC_INTEGRIN_PATHWAY
KE_CITRATE_CYCLE_TCA_CYCLE
KE_ALANINE_ASPARTATE_AND_GLUTAMATE_METABOLISM
KE_SELENOAMINO_ACID_METABOLISM
KE_PEROXISOME
ZT0 ZT4 ZT8 ZT12 ZT16 ZT20 ZT0
ZT0 ZT4 ZT8 ZT12 ZT16 ZT20 ZT0
Z−score
−2 −1 0 1 2

Supplementary Figure 6: Mean expression of dryR-identified transcription factors (TFs) with uniquely rhythmic gene expression pattern in human and/or mouse cells in non-infected control HLCM livers. n=5 HLCM/timepoint. ZT0: represented twice in each panel to maintain conformity. Source data are provided as a Source Data file.

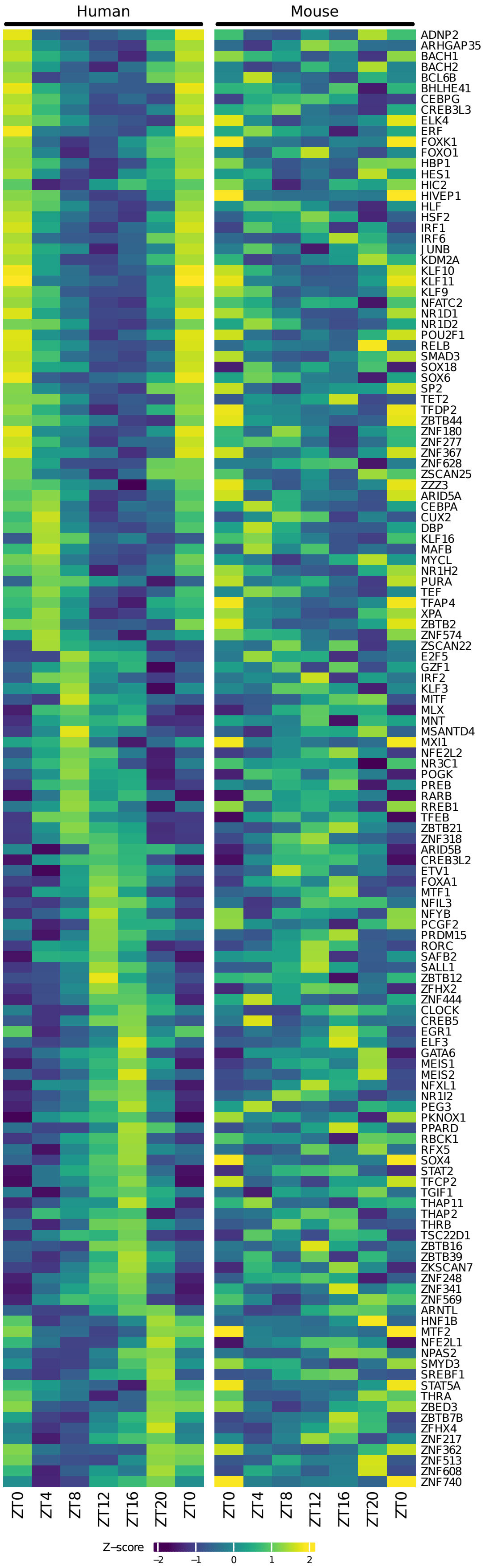

<u>Supplementary Figure 7:</u> Examples of transcription factors (TFs) with rhythmic mean gene expression pattern in human (red) and mouse (green) cells in non-infected control HLCM livers (Fig. 1e) with shifted expression pattern (dryR model 5) shown in **a**, and similar expression pattern (dryR model 4) shown in **b**. Curves are shown as normalized DESeq2 read counts (n=5 HLCM/timepoint). Bars represent SD. Source data are provided as a Source Data file.

a

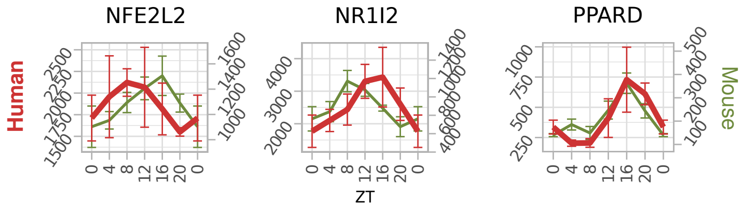

b

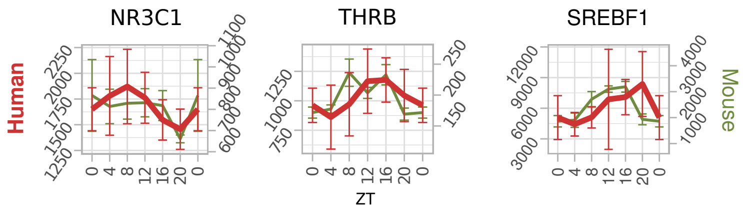

<u>Supplementary Figure 8:</u> ChIP-seq coverage plots indicate temporal variations in H3K27ac levels in the CC-output regulator gene DBP in human hepatocytes from non-infected control HLCM livers (green) and WT mouse livers (red) as published in Koike et al. Science 2012.

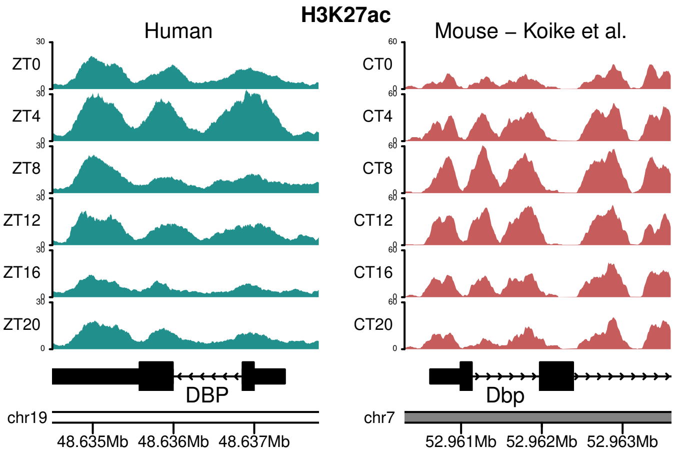

<u>Supplementary Figure 9:</u> Unsupervised clustering of human hepatocytes in HCV-infected HLCM livers and CC genes from two independent experiments: **a** Series 1 and **b** Series 2 (3 HLCM/timepoint, n=18 HLCM/ experiment). Samples highlighted with bold font in **a** were selected for further analysis. Source data are provided as a Source Data file.

a

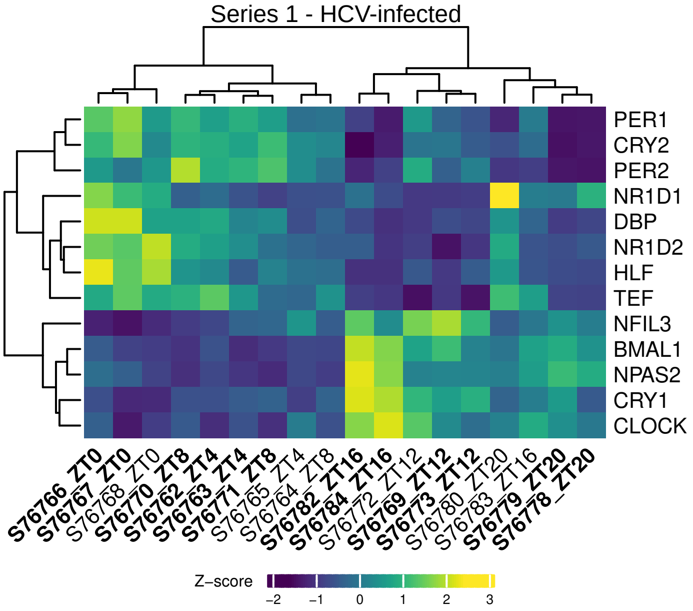

b

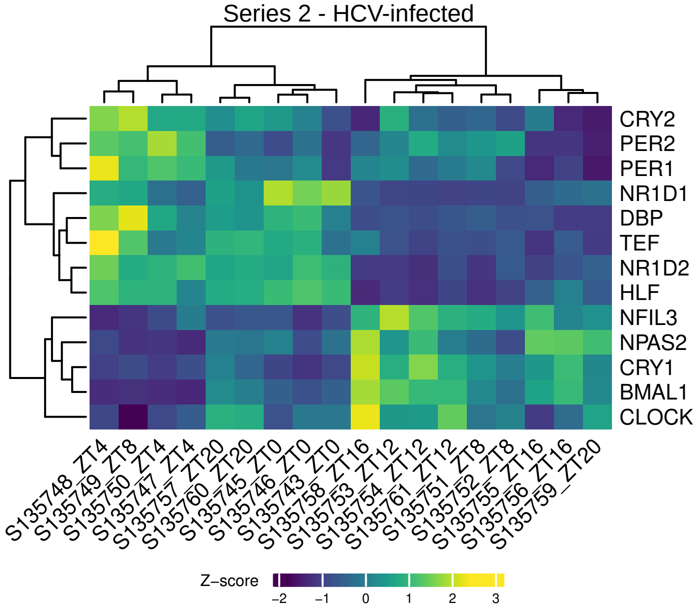

Supplementary Figure 10: **a** Quantification of human-specific CK8-18 staining reveals degree of humanization in non-infected control (grey) and HCV-infected (red) HLCM livers. Each data point represents individual HLCM. All the mice from each group were used for staining and quantification. n=18/condition, Mann-Whitney test, two-tailed, p=0.0001. The box plots show the median (line), the 25th and 75th percentiles (box), and min-to-max values (whiskers). Source data are provided as a Source Data file. **b** Representative images of CK8-18 staining of engrafted human hepatocytes in non-infected control and HCV-infected HLCM livers. The black arrows indicate perivascular regions with unstained murine hepatocytes. Scale bar: 250 µm. **c** Median serum levels (ng/mL; x10^7) of human albumin in control (black line) and HCV-infected (green line) HLCM, as indicated. n=18/condition and timepoint except for control baseline (n=16) and control week 2 (n=16). Mann-Whitney test, two-tailed, see source file for specific p-values. *: p<0.05, **: p<0.01, ***: p<0.001. Source data are provided as a Source Data file.

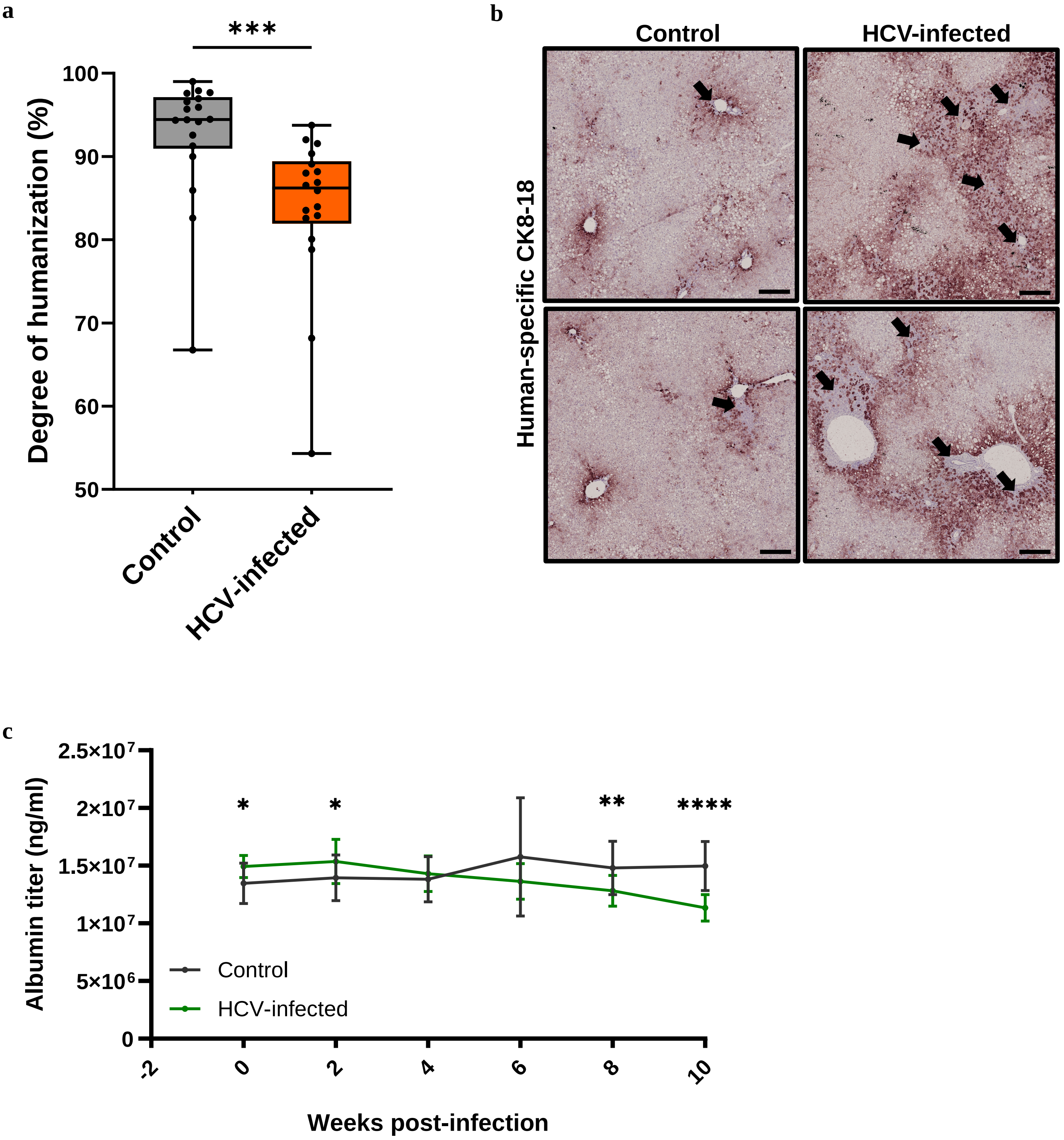

Supplementary Figure 11: Mean serum levels of HCV load in infected HLCM livers at different timepoints, as indicated. n=3 HLCM/timepoint. Source data are provided as a Source Data file.

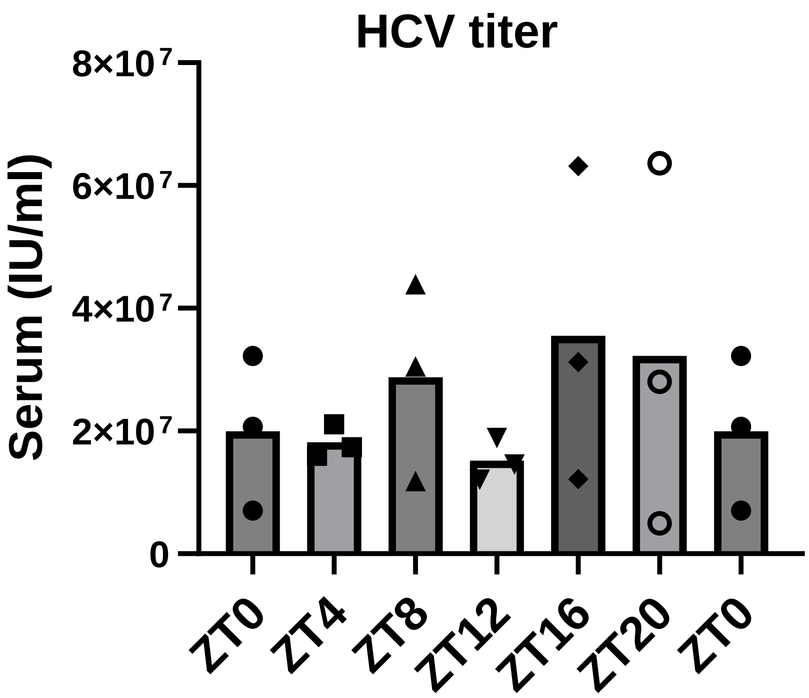

Supplementary Figure 12: Percentage of distinct categories of cycling genes (obtained through dryR analysis) in rhythmic pathways in HCV-infected vs. non-infected control HLCM livers. n=5 HLCM/group and timepoint. Source data are provided as a Source Data file.

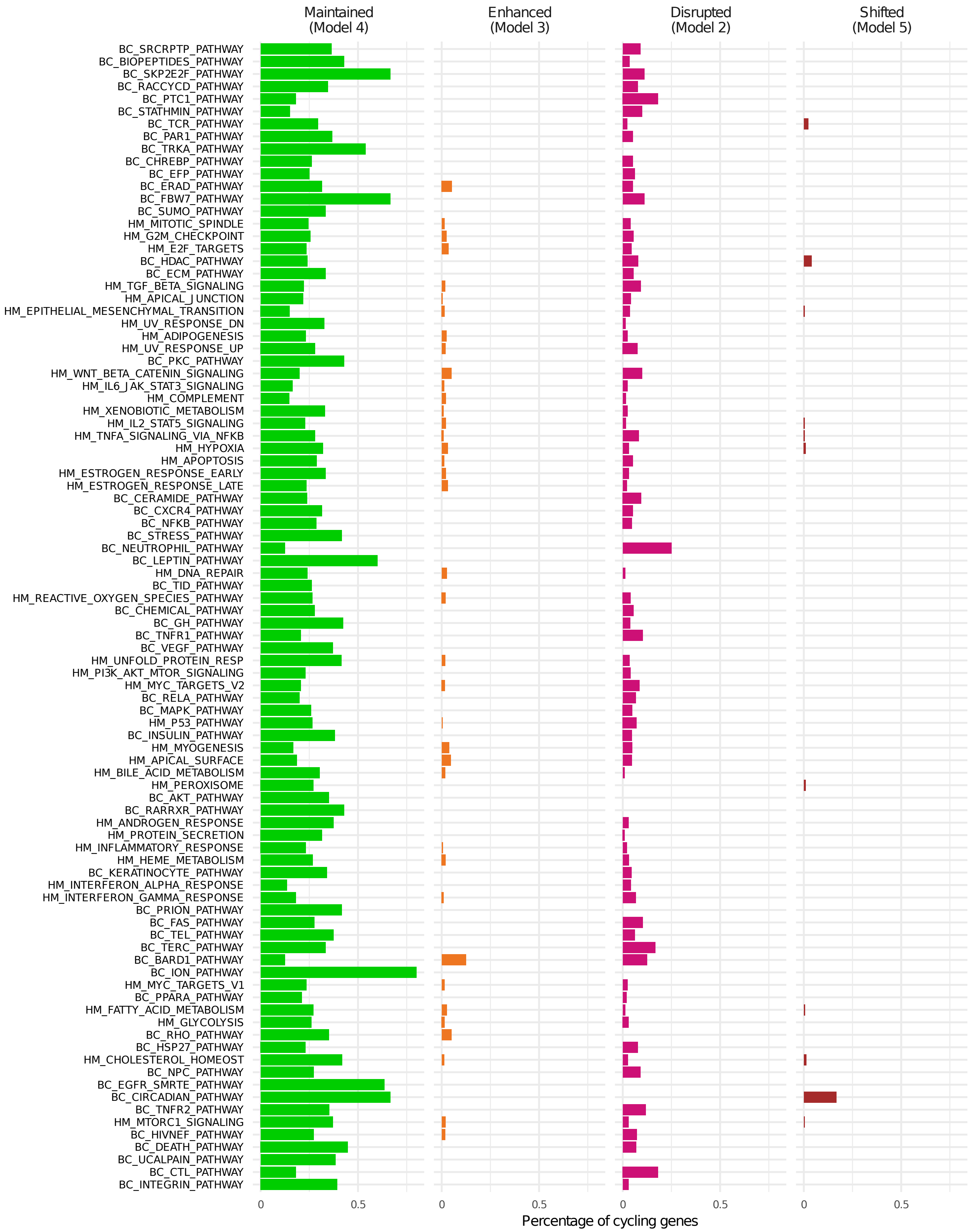

Supplementary Figure 13: Representative images of MYC immunohistochemistry (IHC) from non-infected control (**a**) and HCV-infected (**b**) HLCM livers at indicated timepoints. Note that MYC (oncogene) is barely expressed in control conditions. Scale bar: 300µm. **c** Quantification (mean) of MYC-immuno-stained cells from non-infected control (black/grey) and HCV-infected (red) HLCM livers (as shown in panels **a** and **b**). Bars in line plot represent SD. *: p=0.01557 (Mann-Whitney test, two-tailed, n=18/cindition). The box plots show the median (line), the 25th and 75th percentiles (box), and min-to-max values (whiskers). Source data are provided as a Source Data file.

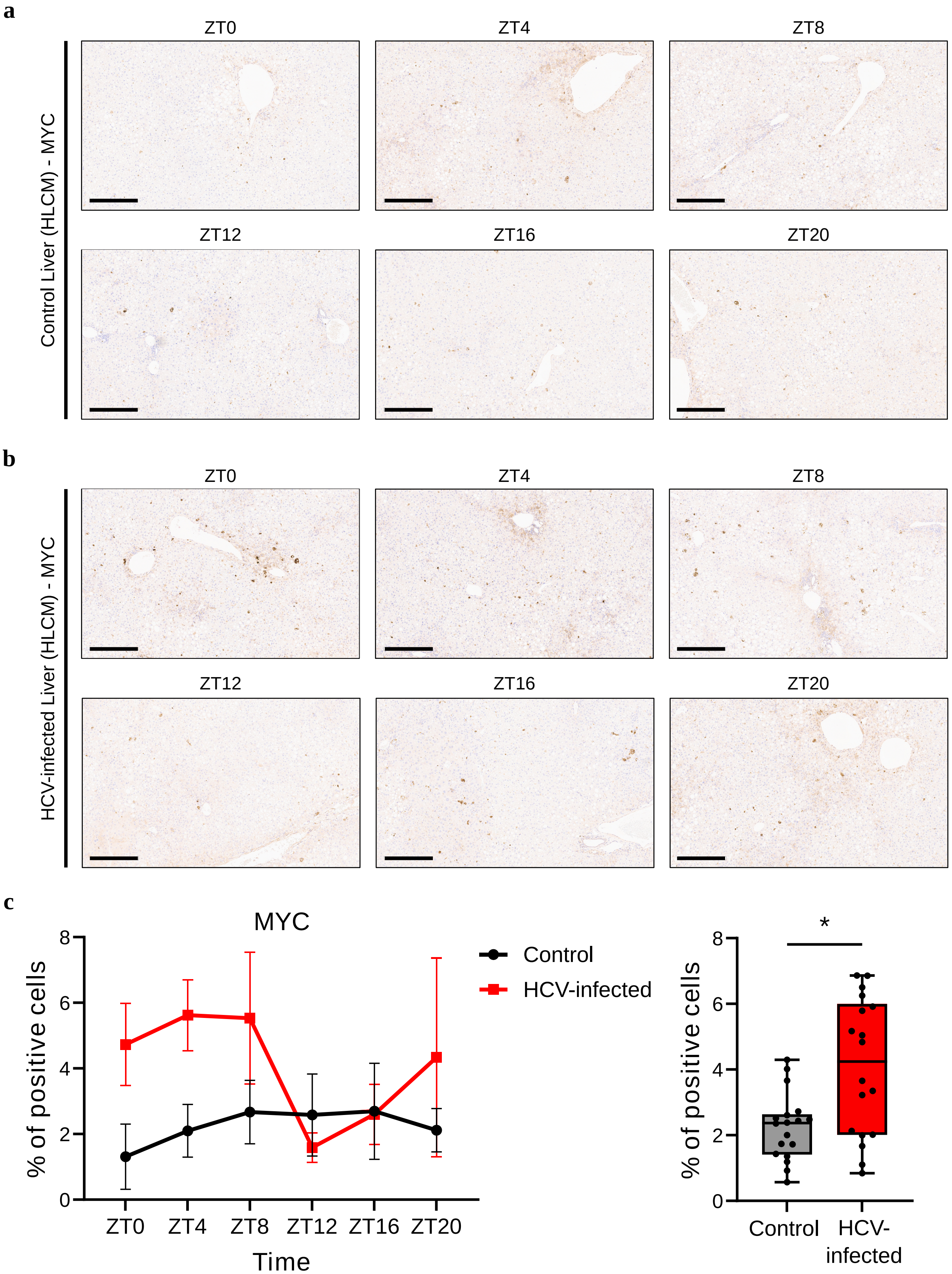

Supplementary Figure 14: **a** Representative images of HE stained liver sections of non-infected control (grey) and HCV-infected (red) HLCM livers, and quantification of the steatosis (inflammation and ballooning). Each point represents an HLCM. Mann-Whitney test, two-tailed, n=18/condition. Source data are provided as a Source Data file. **b** Sirius red staining of the non-infected control and HCV-infected HLCM livers at different diurnal time points indicate mild fibrosis upon viral infection. **c** Quantification of the collagen positive area (CPA) from Sirius red-stained (**b**) in non-infected control (n=18; grey) and HCV-infected (n=18; red) HLCM livers. Mann-Whitney test, two-tailed, n=18/condition, $p<0.0001$. Source data are provided as a Source Data file. The box plots in **a** and **c** show the median (line), the 25th and 75th percentiles (box), and min-to-max values (whiskers).

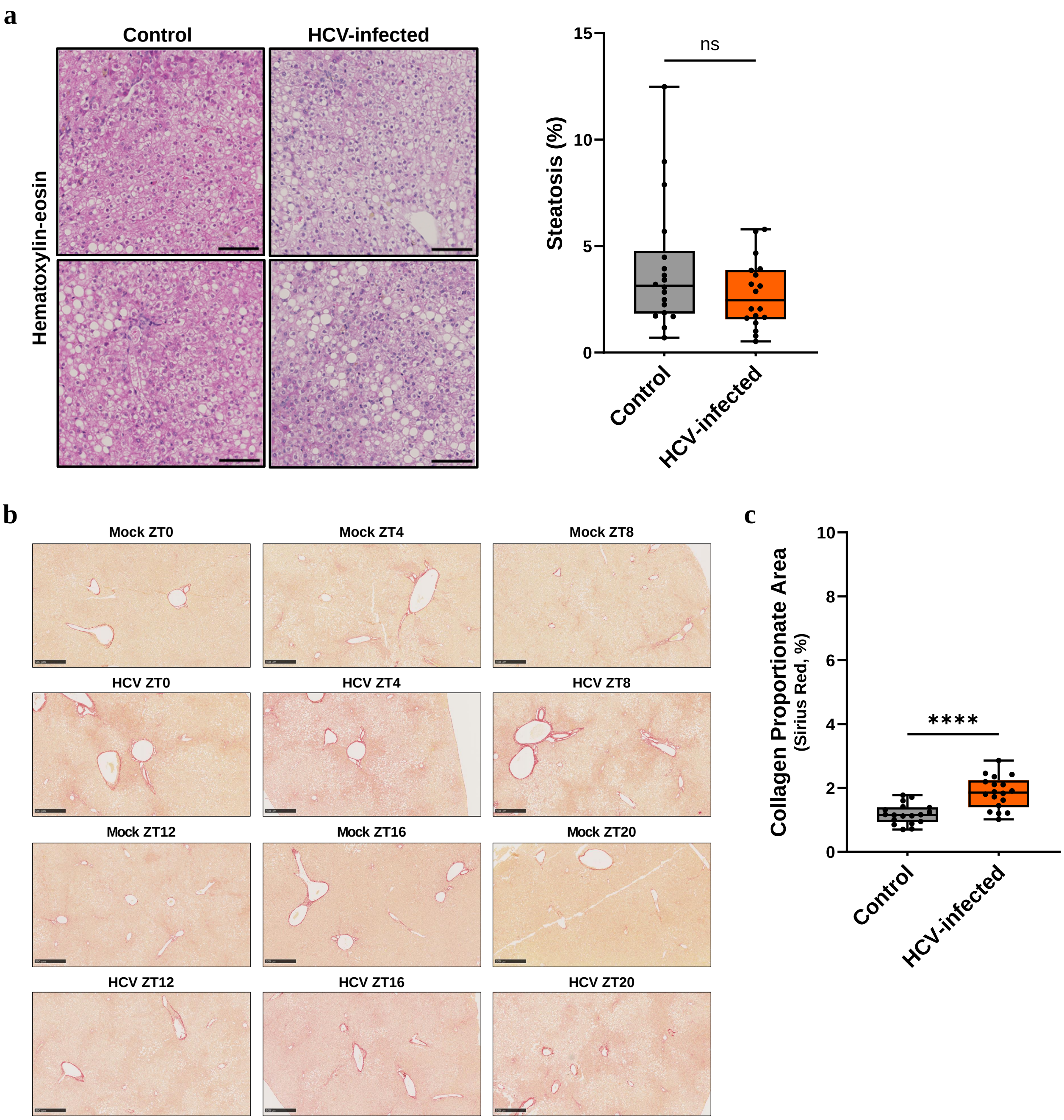

<u>Supplementary Figure 15:</u> **a** Temporal variations in H3K27ac levels in non-infected control (green) and HCV-infected (red) HLCM livers for CRY1. **b** Temporal variations in H3K9ac levels in non-infected control (green) and HCV-infected (red) HLCM livers, for indicated genes.

a

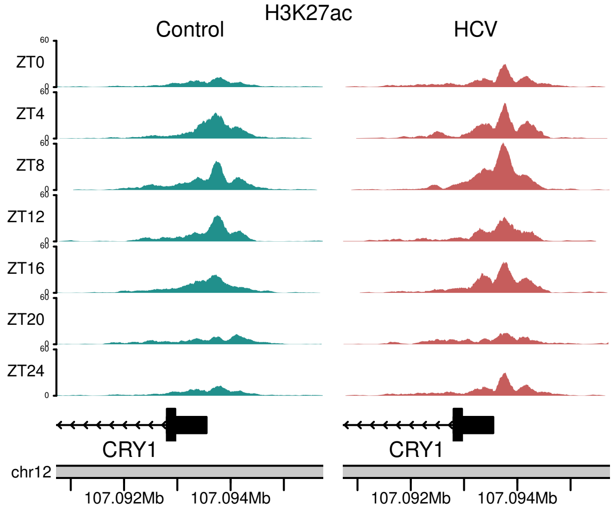

b

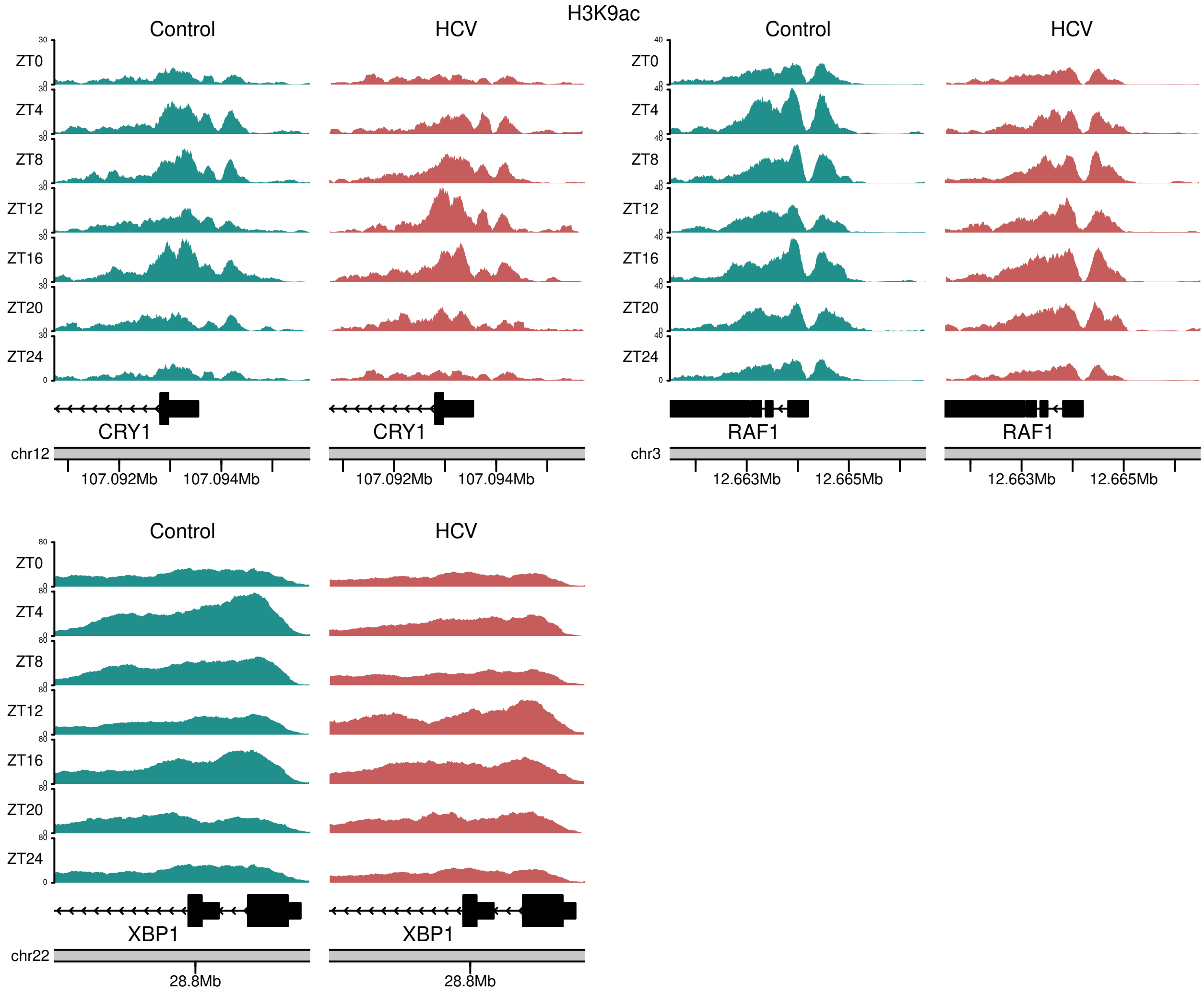

<u>Supplementary Figure 16:</u> a Mean expression pattern of indicated histone acetylases and deacetylases from non-infected control and HCV-infected HLCM livers. The Y-axis represents the DESeq2 normalized reads (n=5 HLCM/group and timepoint). Bars represent SDs. Wald test revealed non-significant differences in expression for all the genes. Source data are provided as a Source Data file. **b** Mean expression pattern of indicated Sirtuin genes in non-infected control and HCV-infected HLCM livers. The Y-axis represents the DESeq2 counts (n=5 HLCM/group and timepoint). Bars represent SD. Two-tailed Wald test as implemented in DESeq2 revealed no significant differences in expression for the indicated genes at all timepoints except for SIRT3 at ZT20 (FDR=0.0495567189) and SIRT5 at ZT20 (FDR=0.0003535596). Source data are provided as a Source Data file.

a

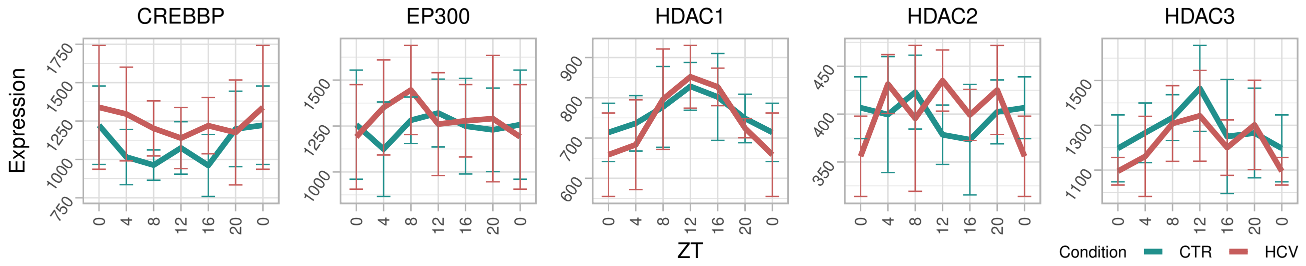

b

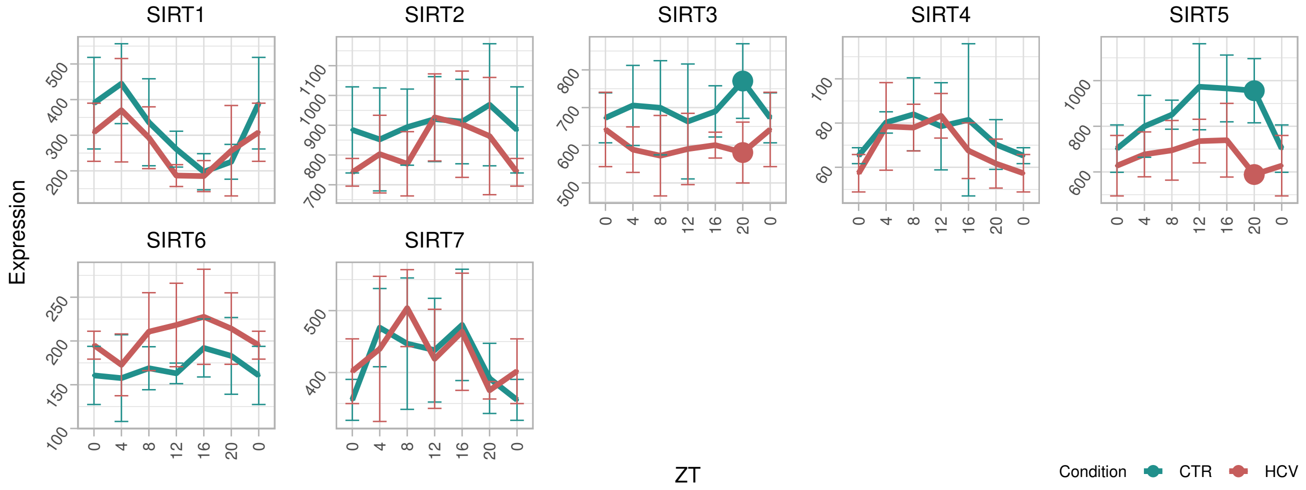

Supplementary Figure 17: **a** Hallmark pathways enriched for human rhythmic genes as identified in Fig. 1d and the prognostic liver signature (PLS; Hoshida et al. N Engl J Med 2008) gene sets in HLCM livers based on the transcriptomic data described in Hamdane et al. Gastroenterology 2019 (shown are gene sets in HCV-infected vs. non-infected control and DAA-cured vs. non-infected control animals). Red=significant (FDR<0.05) enrichment, blue=significant (FDR<0.05) negative enrichment, white=no significant enrichment, unchanged. Source data are provided as a Source Data file. **b** Hallmark pathways enriched for human rhythmic genes as identified in Fig. 1d and the PLS (Hoshida et al. N Engl J Med 2008) gene sets in livers of DAA-cured vs. non-infected control patients based on the transcriptomic data as described in Hamdane et al. Gastroenterology 2019. Samples were obtained from liver tissues adjacent to tumors. Red=significant (FDR<0.05) enrichment, blue=significant (FDR<0.05) negative enrichment, white=no significant enrichment, unchanged. Source data are provided as a Source Data file. **c** Hallmark pathways enriched for human rhythmic genes as identified in Fig. 1d and the PLS (Hoshida et al. N Engl J Med 2008) gene sets in paired liver tissue samples from the same patient (pre- and post-cure) of HCV-infected and DAA-treated patients with advanced liver disease and HCC (Japanese cohort). Red=significant (FDR<0.05) enrichment, blue=significant (FDR<0.05) negative enrichment, white=no significant enrichment, unchanged. Source data are provided as a Source Data file. **d** Hallmark pathways enriched for human rhythmic genes as identified in Fig. 1d and the PLS (Hoshida et al. N Engl J Med 2008) gene sets in the livers of MASH vs. control patients from the transcriptomic data described in Jühling et al. Gut 2021. Red=significant (FDR<0.05) enrichment, blue=significant (FDR<0.05) negative enrichment, white=no significant enrichment, unchanged. Source data are provided as a Source Data file.

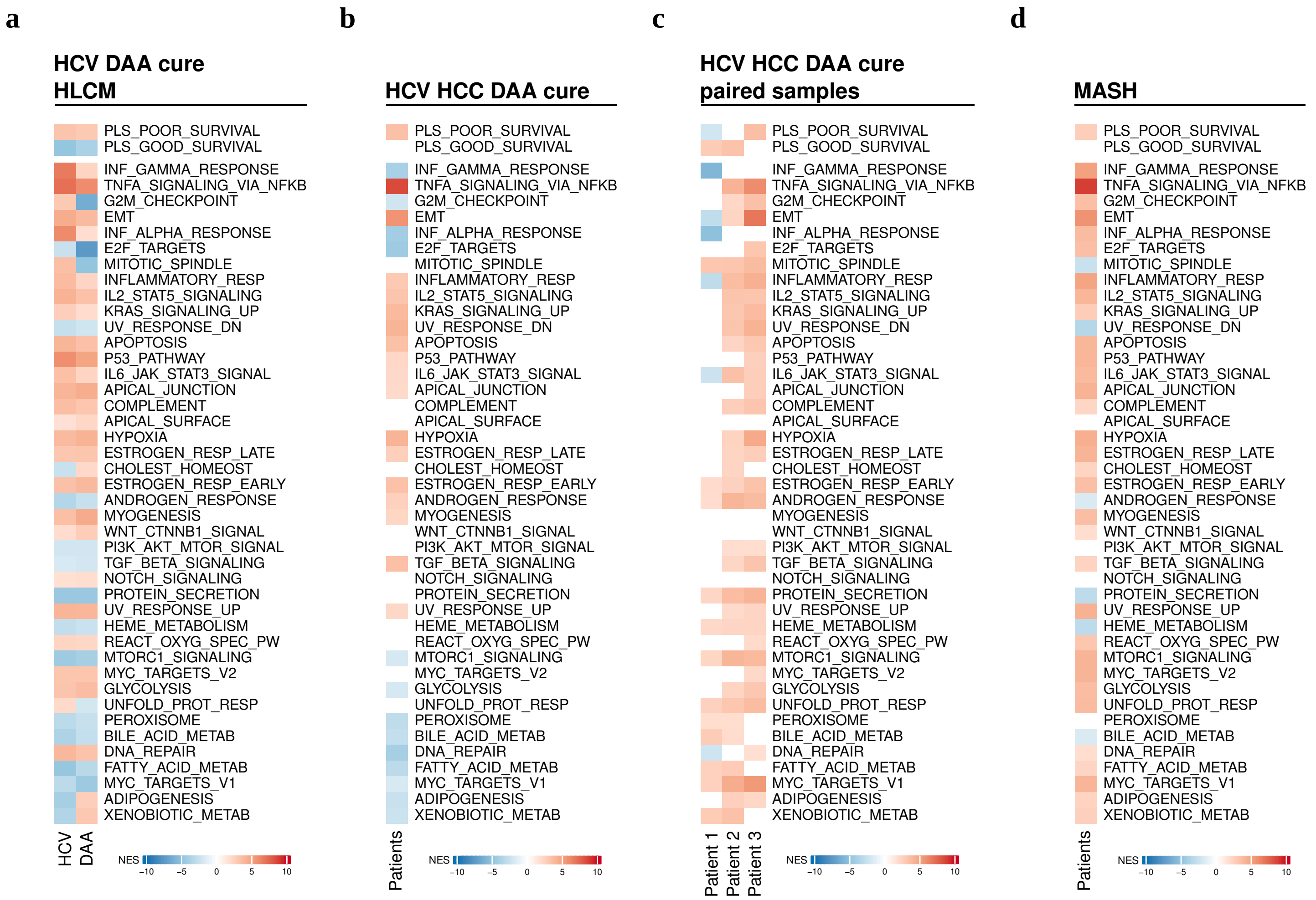

Supplementary Figure 18: Proportions of rhythmic genes of the 186-gene PLS (Hoshida et al. N Engl J Med 2008) and its perturbations by HCV-infection in HLCM livers. Source data are provided as a Source Data file.

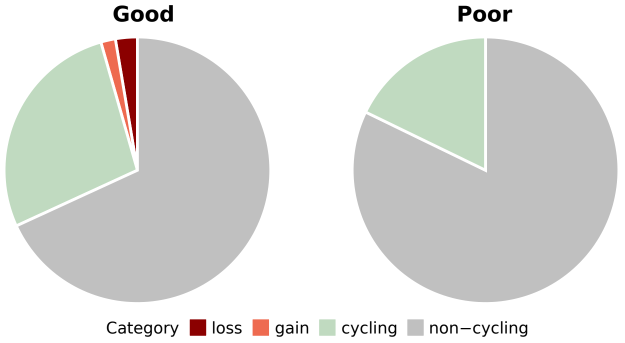

Supplementary Table 1: Demographic and clinical characteristics of HCV-infected and DAA-cured patients.

Patient	ID	Age	Gender	Ethnicity	HCV GT	HCV RNA: log10 IU/ml	HIV	HBV	LC	Pre-CM	Post-CM	DAA regimen
Patient1	Pat1	76	female	Japanese	1b	6.9	0	0	0	2	2	DCV+ASV
Patient2	Pat2	81	male	Japanese	2b	6.1	0	0	0	2	2	SOF+RBV
Patient3	Pat3	67	male	Japanese	1b	6.2	0	0	0	3	2	DCV+ASV

CM, cure Metavir (F score); GT, genotype; DCV, Daclatasvir; ASV, Asunaprevir ; SOF, Sofosbuvir; RBV, Ribavirin.

Supplementary Table 2: Correlation of the CC-oscillator gene signature induction with clinical/molecular phenotypes in 216 HCV cirrhosis patients. Early time points: ZT0-ZT8, late time points: ZT12-ZT20. Two-tailed test p-values and FDRs were calculated.

CC-oscillator gene signature	Clinical/molecular variable	Rho	p-value	FDR
- Gene signatures derived from HCV-infected hepatocytes				
Gain (model 3), early time points	PLS	0.26	0.000	0.007
Loss (model 2), late time points	PLS	0.21	0.002	0.053
- Gene signatures derived from control hepatocytes				
Loss (model 2), early time points	PLS	0.32	0.000	0.000
Loss (model 2), early time points	AST	0.22	0.001	0.020
Gain (model 3), early time points	PLS	0.22	0.001	0.021
Altered (model 5), early time points	Bilirubin	-0.26	0.000	0.006
Altered (model 5), early time points	AST	0.23	0.001	0.013
Unaltered (model 4), early time points	Male sex	0.17	0.015	0.188

CC, circadian clock; PLS, Prognostic Liver Signature; AST, aspartate aminotransferase; AFP, alpha-fetoprotein; FDR, false discovery rate.
