# Supplementary material for: An atlas of the human liver diurnal transcriptome and its perturbation by hepatitis C virus infection
Source: Nat Commun. 2024 Aug 29;15:7486. doi: 10.1038/s41467-024-51698-8 (PMC11362569; doi:10.1038/s41467-024-51698-8)
Supplement: Supplementary file 3 — Description of Additional Supplementary Files [file 41467_2024_51698_MOESM3_ESM.pdf]

Description of Additional Supplementary Files

File Name: Supplementary Data 1
Description: Sample characteristics Series 1.

File Name: Supplementary Data 2
Description: Sequencing characteristics Series 1.

File Name: Supplementary Data 3
Description: Cycling genes: their association with dryR models and the cycling transcriptome as reported in external datasets.

File Name: Supplementary Data 4
Description: The association of human genes with specific dryR models comparing HCV-infected and non-infected control HLCM livers.

File Name: Supplementary Data 5
Description: Sequencing characteristics and peak calling statistics for the H3K27ac dataset.

File Name: Supplementary Data 6
Description: CC-oscillator signatures: genes with > 4-fold over-expression.
