# Supplementary material for: An atlas of the human liver diurnal transcriptome and its perturbation by hepatitis C virus infection
Source: Nat Commun. 2024 Aug 29;15:7486. doi: 10.1038/s41467-024-51698-8 (PMC11362569; doi:10.1038/s41467-024-51698-8)
Supplement: Supplementary file 10 — Reporting Summary [file 41467_2024_51698_MOESM10_ESM.pdf]

nature portfolio

Corresponding author(s): Thomas BAUMERT
Joachim LUPBERGER

Last updated by author(s): Jul 31, 2024

Reporting Summary

Nature Portfolio wishes to improve the reproducibility of the work that we publish. This form provides structure for consistency and transparency in reporting. For further information on Nature Portfolio policies, see our Editorial Policies and the Editorial Policy Checklist.

Statistics

For all statistical analyses, confirm that the following items are present in the figure legend, table legend, main text, or Methods section.

n/a	Confirmed	
☐	☒	The exact sample size (*n*) for each experimental group/condition, given as a discrete number and unit of measurement
☐	☒	A statement on whether measurements were taken from distinct samples or whether the same sample was measured repeatedly
☐	☒	The statistical test(s) used AND whether they are one- or two-sided<br>*Only common tests should be described solely by name; describe more complex techniques in the Methods section.*
☒	☐	A description of all covariates tested
☐	☒	A description of any assumptions or corrections, such as tests of normality and adjustment for multiple comparisons
☐	☒	A full description of the statistical parameters including central tendency (e.g. means) or other basic estimates (e.g. regression coefficient) AND variation (e.g. standard deviation) or associated estimates of uncertainty (e.g. confidence intervals)
☐	☒	For null hypothesis testing, the test statistic (e.g. *F*, *t*, *r*) with confidence intervals, effect sizes, degrees of freedom and *P* value noted<br>*Give P values as exact values whenever suitable.*
☒	☐	For Bayesian analysis, information on the choice of priors and Markov chain Monte Carlo settings
☒	☐	For hierarchical and complex designs, identification of the appropriate level for tests and full reporting of outcomes
☐	☒	Estimates of effect sizes (e.g. Cohen's *d*, Pearson's *r*), indicating how they were calculated

Our web collection on statistics for biologists contains articles on many of the points above.

Software and code

Policy information about availability of computer code

Data collection	MSigDB (7.2), JASPAR (2020)
Data analysis	HCS (HD3.4.0.38), Real time analysis software (2.7.7), Picard tools (2.19.2), bcl2fastq(2.20.0.422) , HiSat2 (2.0.4), R (4.3.3), bowtie (1.2.2), DESeq2 (1.28.1), MACS3 (3.0.0b3), dryR (1.0.0), GSEA (4.3.2), GSVA(1.50.2), karyoploteR(1.28.0), ggplot2(3.5.0), HTSeq (2.0.3), ComplexHeatmap (2.18.0), FastQC (0.12.1)

For manuscripts utilizing custom algorithms or software that are central to the research but not yet described in published literature, software must be made available to editors and reviewers. We strongly encourage code deposition in a community repository (e.g. GitHub). See the Nature Portfolio guidelines for submitting code & software for further information.

Data

Policy information about availability of data

All manuscripts must include a data availability statement. This statement should provide the following information, where applicable:

- Accession codes, unique identifiers, or web links for publicly available datasets
- A description of any restrictions on data availability
- For clinical datasets or third party data, please ensure that the statement adheres to our policy

Raw files and technical details about the RNA-seq and ChIP-seq data have been deposited in the National Center for Biotechnology Information’s Gene Expression Omnibus (GEO) and are accessible through GEO series accession number GSE200812. Raw files for the RNA-seq of the Taiwanese patient cohort (6 HCV-infected

and 6 paired-cured) samples are available on request from Prof. Ray T Chung (Harvard Medical School, USA). Previously published data (GSE84346, SRP170244, PRJNA606244, and GSE 15654) were also used for this work.

Research involving human participants, their data, or biological material

Policy information about studies with human participants or human data. See also policy information about sex, gender (identity/presentation), and sexual orientation and race, ethnicity and racism.

Reporting on sex and gender	Sex and gender of tissue donors is reported in the material section and in the supplementary information.
Reporting on race, ethnicity, or other socially relevant groupings	Has been mentioned in the supplementary information.
Population characteristics	Population characteristics have been provide in the supplementary information.
Recruitment	not applicable
Ethics oversight	Retrospective study. The ethical approval of the respective centers have been indicated in the methods section.

Note that full information on the approval of the study protocol must also be provided in the manuscript.

Field-specific reporting

Please select the one below that is the best fit for your research. If you are not sure, read the appropriate sections before making your selection.

☒ Life sciences ☐ Behavioural & social sciences ☐ Ecological, evolutionary & environmental sciences

For a reference copy of the document with all sections, see nature.com/documents/nr-reporting-summary-flat.pdf

Life sciences study design

All studies must disclose on these points even when the disclosure is negative.

Sample size	No sample size calculations were performed as SVR patients with sequential liver samples are extremely rare. Thus we included as many patient samples as possible. For humanized mice, we performed 2 independent experiments, and each with 3 control and 3 HCV-infected mice were used per circadian time point.
Data exclusions	One mouse (from experiment series 1) was excluded from the subsequent analyses due to a low read count in the RNA-seq. This has been mentioned in the manuscript and nevertheless provided the excluded mouse data in the supplemental information.
Replication	Humanized mice study was performed twice, independently. Patient samples being rare we consolidated findings in additional cohorts as described in manuscript.
Randomization	Humanized mice were randomly assigned for control and HCV infection.
Blinding	Investigators were blinded to group allocation during processing of samples for RNA-seq, ChIP-seq and Immunohistochemistry from control and HCV-infected mice.

Reporting for specific materials, systems and methods

We require information from authors about some types of materials, experimental systems and methods used in many studies. Here, indicate whether each material, system or method listed is relevant to your study. If you are not sure if a list item applies to your research, read the appropriate section before selecting a response.

Materials & experimental systems

n/a	Involved in the study	
☐	☒	Antibodies
☒	☐	Eukaryotic cell lines
☒	☐	Palaeontology and archaeology
☐	☒	Animals and other organisms
☐	☒	Clinical data
☒	☐	Dual use research of concern
☒	☐	Plants

Methods

n/a	Involved in the study	
☐	☒	ChIP-seq
☒	☐	Flow cytometry
☒	☐	MRI-based neuroimaging

Antibodies

Antibodies used	ChIP-seq was conducted using antibodies targeting H3K27ac (Active Motif; #39133), H3K9ac (Active Motif, #39137) and IgG (Diagenode, #C15410206). Following antibodies were used for IHC: CK18 (CK18, M701029-2, Clone DC 10, Dako), and MYC (Abcam, #ab32072).
Validation	Vendors provided application notes for their validation of antibodies.

Animals and other research organisms

Policy information about studies involving animals; ARRIVE guidelines recommended for reporting animal research, and Sex and Gender in Research

Laboratory animals	uPA/SCID mice were obtained from PhenixBio: "PXB-mouse" [Genotype/Strain: cDNA-uPAwild/+/SCID, cDNA-uPAwild/+: B6;129SvEv-Plau, SCID: C.B-17/Icr-scid/scid Jcl]
Wild animals	Not applicable
Reporting on sex	All animal have been male as liver cancer develops predominantly in this sex. This has been specified in the manuscript.
Field-collected samples	Not applicable.
Ethics oversight	Ethical statement has been provided in the manuscript.

Note that full information on the approval of the study protocol must also be provided in the manuscript.

Clinical data

Policy information about clinical studies

All manuscripts should comply with the ICMJE guidelines for publication of clinical research and a completed CONSORT checklist must be included with all submissions.

Clinical trial registration	Not applicable
Study protocol	Not applicable
Data collection	Not applicable
Outcomes	Not applicable

Plants

Seed stocks	Not applicable
Novel plant genotypes	Not applicable
Authentication	Not applicable

ChIP-seq

Data deposition

☒ Confirm that both raw and final processed data have been deposited in a public database such as GEO.

☒ Confirm that you have deposited or provided access to graph files (e.g. BED files) for the called peaks.

Data access links<br>*May remain private before publication.*	GSE200812 [https://www.ncbi.nlm.nih.gov/geo/query/acc.cgi?acc=GSE200812]
Files in database submission	Raw reads (fastq files) and called peaks (narrowPeak files)

Genome browser session (e.g. UCSC)	Not applicable

Methodology

Replicates	No replicates; one sample from control and one sample from HCV per circadian timepoint was used for ChIP-seq.
Sequencing depth	Reads are single-end paired and with a length of 50; see Supplementary Data File 5 for details on sequencing depth.
Antibodies	H3K27ac antibody (39133, Active motif), H3K9ac antibody (39137, Active motif), and control Rabbit IgG (C15410206, Diagenode).
Peak calling parameters	All original Peaks were called in uniquely mapped reads using MACS3. See Supplementary Data File 5 for details about peak calling.
Data quality	Quality control checks were performed using FastQC
Software	We used bowtie for mapping down-sampled all alignments to similar read numbers, and applied macs3 with standard parameters and corresponding inputs for peak calling.
